# Integrative research identifies 71 new plant species records in the state of Rio Grande do Norte (Brazil) and enhances a small herbarium collection during a funding shortage

**DOI:** 10.3897/phytokeys.86.13775

**Published:** 2017-09-18

**Authors:** Leonardo M. Versieux, Nállarett Dávila, Geadelande C. Delgado, Valdeci F. de Sousa, Edweslley Otaviano de Moura, Tarciso Filgueiras, Marccus V. Alves, Eric Carvalho, Daniel Piotto, Rafaela C. Forzza, Alice Calvente, Jomar G. Jardim

**Affiliations:** 1 Universidade Federal do Rio Grande do Norte, Centro de Biociências, Departamento de Botânica e Zoologia, Campus Universitário, Lagoa Nova, Natal, RN, 59.072-970, Brazil; 2 Serviço Florestal Brasileiro, SCEN, Av. L4, Trecho 2, Bloco H, Brasília, DF, 70.818-900, Brazil; 3 Instituto de Botânica, Av. Miguel Stéfano 3687, Água Funda, São Paulo, SP, 04.301-902, Brazil; 4 Universidade Federal de Pernambuco, Laboratório de Morfo-Taxonomia Vegetal, Av. Moraes Rego s.n., CDU, Recife, PE, 50.670-901, Brazil; 5 Programa de Pós-graduação em Botânica, Universidade Federal Rural de Pernambuco, Recife, PE, 52.171-900, Brazil; 6 Jardim Botânico do Rio de Janeiro, Rua Pacheco Leão 915, Rio de Janeiro, RJ, 22.460-030, Brazil; 7 Universidade Federal do Sul da Bahia, Instituto de Humanidades, Artes e Ciências, Campus Jorge Amado, Rod. BR 415, Ferradas, Itabuna, BA, 45613-204, Brazil

**Keywords:** Brazil Flora Group, Flora, floristics, Herbarium collection, Inventory, Reflora, SiBBr, IFN

## Abstract

A National Forest Inventory (NFI) encompassing the entire territory of Brazil is in progress. It is coordinated and promoted by the Brazilian Forest Service of the Ministry of Environment. In each state, the NFI collaborates with local herbaria by receiving collected plant material and performing species identification. Consultants are hired by the NFI and work at the local herbaria under the supervision of a curator. In exchange for curatorial assistance, the NFI provides equipment and consumables for the herbarium. Other public projects collaborating with NFI are Reflora and the Brazilian Biodiversity Information System (SiBBr). Both projects have online platforms that seek to connect herbaria and make all their data freely available, including high quality digital images of specimens. Through inter-institutional collaboration, the joint interests of NFI, Reflora, SiBBr and local herbaria have improved collections, expanded the online Reflora database, and provided the NFI with verified species lists. These strategic uses of public funding are positively affecting Botany, particularly during a period of economic crisis and cuts in research. Here, we illustrate the increase in floristic knowledge through the improvement of a herbarium collection in Rio Grande do Norte (RN) – the Brazilian state with the lowest levels of plant richness. We report 71 new occurrences of vascular plants for RN, belonging mainly to the Poaceae, Fabaceae and Malvaceae. Most of the species with new occurrences have a Neotropical distribution (21 spp.) and only seven are restricted to the Brazilian Northeast. Our findings highlight previous gaps in RN’s floristic knowledge. The partnership NFI, Reflora, SiBBr and the UFRN herbarium improved herbarium curation, digital collection, and quality of data. Finally, a fellowship provided by Reflora and SiBBr allowed improving curation by distributing duplicates and incorporating the Herbarium of Câmara Cascudo Museum.

## Introduction

A National Forest Inventory (NFI) covering all of Brazil is currently in progress. The NFI is a major undertaking by the Brazilian government, specifically, the Brazilian Forest Service, a public section of the Ministry of Environment, to periodically gather information about the forests and land cover of Brazil, through a systematic sampling of the territory using a 20 km × 20 km grid. In each state, the NFI invites local herbaria to receive and identify the collected specimens. Consultants are hired by the NFI and stay at the local herbarium under the supervision of a curator to identify plants to species. In exchange for this curatorial assistance, the NFI helps the herbarium by providing equipment and consumables. In Rio Grande do Norte (RN), the NFI started in 2014. Two additional public projects that are working with the NFI are Reflora and the Brazilian Biodiversity Information System (SiBBr). Reflora and the SiBBr are online platforms that connect herbaria, making data and high quality images of specimens in their collections freely available. The main goal of Reflora is to complete the Flora do Brasil 2020 online project, which relies on specimen data and images from herbaria in Brazil, the USA, and Europe. Inter-institutional collaboration serves the interests of NFI, Reflora, SiBBr and local herbaria, improving collections, expanding the Reflora database, and providing the NFI with accurate lists of plants. In this paper, we discuss the details and results of a four-part collaboration that makes strategic use of public funding to positively impact the study of Botany during tough economic times.

Rio Grande do Norte (RN) is a Brazilian state that consists of two phytogeographical domains: Dry Woodlands (Caatinga) and Atlantic Forest (Floresta Atlântica). The savanna (Cerrado) vegetation is scattered in small patches throughout the state. Different vegetation types occur within these phytogeographical domains: deciduous, semi-deciduous, subperennial and seasonal mixed palm forest (dominated by *Copernicia prunifera* (Mill.) H.E. Moore), dunes and coastal sand plain vegetation (restinga), xeric rocky outcrops, natural and anthropic fields, mangroves, saline desert and aquatic vegetation (SUDENE 1971). Both of the main phytogeographical domains in RN have been profoundly altered by human activities. The Atlantic Forest, where most of the sugar cane cultivation has been done for centuries, is fragmented and degraded and urgently needs ecological restoration. Its remaining coverage varies from 8–17%, depending on whether mangroves and restinga coastal plains are included or excluded in the estimate (Maciel et al. 2011). The Caatinga has lost 45% of its original coverage in RN (C. Fonseca, Dept. Ecology, UFRN, pers. com.); what remains is within a few protected areas.

The *Lista de Espécies da Flora do Brasil* (Forzza et al. 2010, 2012) and the *Checklist das plantas do Nordeste Brasileiro* (Barbosa et al. 2006) gathered preliminary knowledge of the flora of RN. Recent taxonomic work has complemented this knowledge by focusing on specific taxonomic groups or on floristic studies. Groups that have been studied recently include *Chamaecrista* (Queiroz and Loiola 2009), Turneraceae (Rocha et al. 2012), *Paspalum* (Oliveira et al. 2013a), Leguminosae-Papilionoideae (São-Mateus et al. 2013), Erythroxylaceae (Costa-Lima et al. 2014), Capparaceae (Soares-Neto and Jardim 2015), *Cyperus* (Ribeiro et al. 2015a), Fabaceae (Amorim et al. 2016), and Bignoniaceae (Colombo et al. 2016). Recent floristic studies focused on specific areas or vegetation types, such as the deciduous and semi-deciduous forests (Cestaro and Soares 2004; 2008), savanna (Oliveira et al. 2012), riparian vegetation (Oliveira et al. 2013b, Ribeiro et al. 2014) and the herbaceous vegetation in Seridó (Santana and Souto 2006, Amorim et al. 2006, Ferreira et al. 2009, Queiroz et al. 2015). Furthermore, field work in RN has produced new records (e.g. Versieux et al. 2013a, 2013b). It is likely that the historically limited number of herbaria (only two in Index Herbariorum), graduate programs focused on biodiversity, and taxonomists may have led to insufficient sampling and underestimation of the true taxonomic diversity of the state.

The most recent account listed 1,222 species of angiosperms in RN, only five of which are endemic to the state (BFG 2015, *Flora do Brasil 2020*): *Aspilia procumbens* Baker (Asteraceae), *Arachis seridoensis* Valls et al. (Fabaceae), *Sida macaibae* Mont. (Malvaceae), *Eugenia pipensis* A.R.Lourenço & B.S.Amorim (Myrtaceae), and *Gouinia virgata* (J. Presl) Scribn. (Poaceae). The growing knowledge of flora in RN is striking and is illustrated by estimates of species richness. In 2010 the RN list included 707 species of angiosperms (Forzza et al. 2010), while in 2015 this number nearly doubled to 1,222 species – a 73% increase in five years (BFG 2015). Research investments that supported these results include the participation of researchers in inter-institutional projects, the creation of two new graduate programs in biodiversity in the largest university of the state (UFRN), and an increase in the number of botanical monographs. We also expect an increase in the publication of floristic studies in the next few years, since many recently-collected data are still in preparation.

Although the UFRN herbarium is a small collection (~25,000 specimens), it is the most representative of RN’s flora. The objective of this paper is to describe the progress in the RN floristic knowledge after joint efforts dedicated to the NFI in RN. Together, IFN, SiBBr and Reflora projects have been addressing poor species coverage and lack of investments in botanical collections. Having completed this field inventory, we can show the new species records for the RN flora, and whether these species are restricted to the northeast of Brazil or else are widely-distributed species that have been previously overlooked in RN. Finally, we report how participation in this joint initiative has influenced and affected the infrastructural legacy of the UFRN herbarium.

## Material and methods

The NFI fieldwork in RN was carried out from March to October 2014 by private environmental consultants, and specimens began to be deposited in the UFRN herbarium in December 2014. The sampling units of the NFI are distributed according to the National Sampling Points Grid (*Grade Nacional de Pontos Amostrais* – GNPA), established by the Brazilian Forest Service. The grid density is 20×20 km, covering all of Brazil (IFN 2012). A total of 133 sampling units, called conglomerates, were placed systematically throughout RN. A conglomerate is composed of four subunits (20×50 m), which are established in the field following magnetic cardinal directions, and radiating 50 m from a central point. Inside each subunit, representative specimens of each species of herbaceous and woody plants were collected following specific protocols for Caatinga and Atlantic Forest (IFN 2012). Biophysical data including necromass, litter and soil characteristics, as well as socio-environmental data were also collected in each conglomerate (Freitas et al. 2016). Our summaries presented here are based only on the species richness data from conglomerates, as well as on the new occurrences indicated by specialists that visited our collections or received duplicates of material previously collected and deposited in UFRN herbarium.

A total of 556 voucher specimens were collected and analyzed to estimate the number of new occurrences for RN. All specimens collected were identified at the UFRN Herbarium using appropriate taxonomic literature and floras, comparisons with specimens identified by specialists, or direct determination by taxonomic specialists. We also incorporated the collection of the Câmara Cascudo Museum (MCC), which used to be an independent collection within UFRN. The MCC collection was partially revised by a technician provided by Reflora and SiBBr projects. All vouchers from the NFI were deposited at UFRN (including non-fertile material) and duplicates were sent to other herbaria (RB, HUEFS, UFP, SP, MG; acronyms follow Thiers continuously updated). Furthermore, during the project, the entire UFRN herbarium collection was digitalized into high quality images that are now available in Jabot platform http://ufrn.jbrj.gov.br.

We used *Lista de Espécies da Flora do Brasil* (http://floradobrasil.jbrj.gov.br), now updated to *Flora do Brasil 2020*, to determine whether species identified were new records for the state. Though *Flora do Brasil 2020* should be continuously updated, we highlight new records in our list, in case that new occurrences reported in scientific literature have been missed.

To get a better picture of the flora of RN, we checked whether the new occurrences are taxa with broad or restricted ranges, as this information may indicate the degree to which they are absent from collections. We defined five categories of distribution according to geographical and political boundaries to infer whether new species records had a distribution restricted to the northeast of Brazil or else they are more widely distributed: 1. Pantropical (“Cosmopolite”): occurring in many places even outside of the tropics, 2. American: occurring all over the Americas, 3. Neotropical: occurring in the Neotropical region, 4. Brazilian: occurring in many states of Brazil – not exclusive to the Northeast region, 5. Northeast: occurring in the Northeast region of Brazil. In addition, we provide comments about each new recorded taxon, including the phytogeographical domains and municipalities where it occurs in RN. Maps were created in QGIS 2.14 (QGIS Development Team 2016) using TEOW (Terrestrial Ecoregions of the World) as a cartographic base (Olsen et al. 2001).

The Brazilian states are abbreviated as follows: AC: Acre, AL: Alagoas, AP: Amapá, AM: Amazonas, BA: Bahia, CE: Ceará, DF: Distrito Federal, ES: Espírito Santo, GO: Goiás, MA: Maranhão, MT: Mato Grosso, MS: Mato Grosso do Sul, MG: Minas Gerais, PA: Pará, PB: Paraíba, PR: Paraná, PE: Pernambuco, PI: Piauí, RJ: Rio de Janeiro, RN: Rio Grande do Norte, RS: Rio Grande do Sul, RO: Rondônia, RR: Roraima, SC: Santa Catarina, SP: São Paulo, SE: Sergipe, TO: Tocantins.

## Results

**Figure 1.**
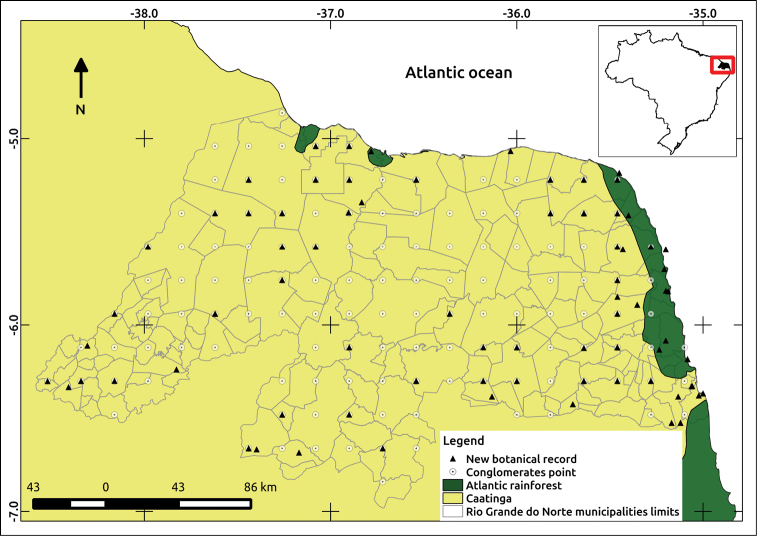
Map of Rio Grande do Norte state and municipalities, phytogeographical domains (Atlantic Rainforest and Caatinga), conglomerates points and new botanical records.

The NFI sampled 133 conglomerates in RN, including 127 in Caatinga and six in Atlantic Rainforest (Figure 1). The sampling covered 86 out of a total of 167 municipalities in the state. We recorded a total of 556 specimens, 285 species and 57 families. The Cerrado was not sampled.

We found 71 newly-occurring species (Table 1) in RN, 43 of which were a result of the NFI inventory (see Fig. 1, overlapping new records and conglomerates points) and 28 of them resulted from additional research developed at the UFRN herbarium and the MCC collection. These new occurrences include species from 21 families, most of them belonging to Poaceae (14 spp.), Fabaceae (13), Malvaceae (13) and Cyperaceae (7). New occurrences have been reported in 55 different municipalities in RN and the municipalities with the highest number of new occurrences were Canguaretama (7 spp.), Ceará-Mirim (6), and Macaíba (4). Most newly-occurring species have a Neotropical distribution (21) and only seven species are restricted to the Northeast of Brazil (Table 2).

Considering the curatorial improvement of the UFRN collection we highlight the merging of the Museu Câmara Cascudo into UFRN herbarium. The specimens from the Museu Câmara Cascudo included 405 angiosperms (54 families), and 1,224 macroalgae (31 families). Algae were not studied in this work, but we indicate the new occurrences among angiosperms (Table 2).

**Table 1.** List of new floristic records of the National Forest Inventory from Rio Grande do Norte state, Brazil. *Sterile specimen **Species previously cited in the literature but not in *Flora do Brasil 2020*.

Family	Species	Voucher	Municipality	Distribution
Amaranthaceae	*Froelichia humboldtiana* (Roem. & Schult.) Seub.	Silva, A.F. 43; Moura, E.O. 125	Serra do Mel; Timbaúba dos Batistas	Neotropical
Anacardiaceae	*Spondias purpurea* L.	Gonçalves, F.B. 447*	Ceará-Mirim	Neotropical
Asteraceae	*Stilpnopappus laiseae* R.Barros & R.Esteves	Silva, A.F. 54	Serra Negra do Norte	Northeast
Burseraceae	*Protium heptaphyllum* (Aubl.) Marchand	Gonçalves, F.B. 405; Dantas, A. 191	Canguaretama; Touros	Neotropical
Cactaceae	*Melocactus ernestii* Vaupel	Souza, A.C.D. 13	Serra de São Bento	Brazilian
Celastraceae	*Maytenus acanthophylla* Reissek	Jardim, J.G. 6393	Serra de São Bento	Brazilian
Chrysobalanaceae	**Hirtella ciliata Mart. & Zucc.	Gonçalves, F.B. 417; 418; 441; MCC	Touros; Pureza	Neotropical
Chrysobalanaceae	**Hirtella racemosa Lam.	Santos, L.A.S. 1268	Ceará-Mirim	Neotropical
Cyperaceae	*Becquerelia cymosa* Brongn.	Roque, A.A. 1375	Canguaretama	Neotropical
Cyperaceae	*Eleocharis flavescens* (Poir.) Urb.	Jardim, J.G. 5823	Extremoz	American
Cyperaceae	*Eleocharis maculosa* (Vahl) Roem. & Schult.	Jardim, J.G. 5624	Extremoz	American
Cyperaceae	*Eleocharis montana* (Kunth) Roem. & Schult.	Oliveira, A.C.P. 1357; Roque, A.A. 929	Ceará-Mirim; São João do Sabugi	American
Cyperaceae	*Rhynchospora caracasana* (Kunth) Boeckeler	Moura, E.O. 149; Ribeiro, A.R.O. 48	Caraúbas; Canguaretama	Neotropical
Cyperaceae	*Rhynchospora gigantea* Link	Jardim, J.G. 6756	Canguaretama	American
Cyperaceae	*Scleria macrophylla* J.Presl & C.Presl	Jardim, J.G. 6757	Canguaretama	American
Euphorbiaceae	*Bernardia sidoides* (Klotzsch) Müll. Arg.	Dantas, R. 63; Silva, A.F. 20	Currais Novos; Açu	Neotropical
Euphorbiaceae	*Croton campestris* A.St.-Hil.	Gonçalves, F.B. 423	Parazinho	Neotropical
Euphorbiaceae	*Ditaxis malpighiacea* (Ule) Pax & K.Hoffm.	Gonçalves, F.B. 371	Macaíba	Brazilian
Euphorbiaceae	*Manihot esculenta* Crantz	Gonçalves, F.B. 349	Serra Caiada	Pantropical
Fabaceae	**Amburana cearensis (Allemão) A.C.Sm.	Gonçalves, F.B. 465*; Silva, A.F. 30*; Dantas, R. 78*	Campo Redondo; Upanema; Venha Ver	Neotropical
Fabaceae	**Ancistrotropis peduncularis (Kunth) A.Delgado	Silva, A.F. 60	Florânia	Neotropical
Fabaceae	*Bauhinia dubia* G.Don	Moura, E.O. 218	Areia branca	Brazilian
Fabaceae	**Dioclea violacea Mart. ex Benth	Borges, S. 257	Natal	American
Fabaceae	*Calliandra depauperata* Benth.	Dantas, R. 3; 103*	Macau; Mossoró	Brazilian
Fabaceae	*Calliandra sessilis* Benth.	Dantas, R. 102*	Macau	Brazilian
Fabaceae	Inga cf. vera Willd.	Dantas, R. 47A*	Jardim de Piranhas	Neotropical
Fabaceae	**Mimosa invisa Mart. ex Colla	Silva, A.F. 100	Riacho de Santana	Neotropical
Fabaceae	**Parkinsonia aculeata L.	Silva, A.F. 91; Dantas, R. 61; Santos, L.A.S 1246	Pilões; São José do Seridó; Santo Antônio	Pantropical
Fabaceae	**Poincianella bracteosa (Tul.) L.P.Queiroz	Silva, A.F. 5; Dantas, A. 295	Mossoró; Touros	Neotropical
Fabaceae	**Poincianella pyramidalis (Tul.) L.P.Queiroz	Dantas, R. 27; Silva, R.E 7	Governador Dix-Sept Rosado; Caraúbas	Brazilian
Fabaceae	**Stylosanthes humilis Kunth	Dantas, R. 66; Silva, A.F. 93	Currais Novos; Marcelino Vieira	Neotropical
Fabaceae	**Trischidium molle (Benth.) H.E.Ireland	Dantas, R. 33*; Gonçalves, F.B. 431; Santos, L.A.S. 1267	Carnaubais; Ceará-Mirim; Pureza	Brazilian
Lecythidaceae	*Eschweilera ovata* (Cambess.) Mart. ex Miers	Dantas, A. 241	Canguaretama	Brazilian
Malvaceae	*Ayenia erecta* Mart. ex K.Schum.	Moura, E.O. 133	Severiano Melo	northeast
Malvaceae	*Ceiba glaziovii* (Kuntze) K.Schum.	Santos, L.A.S. 1229	Santa Cruz	northeast
Malvaceae	*Helicteres cf. guazumifolia* Kunth	Gonçalves, F.B. 382*	Macaíba	Neotropical
Malvaceae	*Herissantia crispa* (L.) Brizicky	Dantas, R. 39; Silva, A.F. 1002; Silva R.E. 10	Carnaubais; Santa Cruz; Porto do Mangue	American
Malvaceae	*Malachra fasciata* Jacq.	Dantas, A. 215	Tibau do Sul	American
Malvaceae	*Melochia tomentosa* L.	Santos, L.A.S. 1226	Santa Cruz	American
Malvaceae	*Pavonia cancellata* (L.) Cav.	Silva, A.F. 52; Gonçalves 394	Serra Negra do Norte; Lagoa Salgada	American
Malvaceae	**Pseudobombax marginatum (A.St.-Hil., Juss. & Cambess.) A. Robyns	Silva, A.F. 99*	Riacho de Santana	Neotropical
Malvaceae	*Sida acuta* Burm.f.	Gonçalves, F.B. 360	Goianinha	Pantropical
Malvaceae	*Sida ciliaris* L.	Gonçalves, F. B. 397; 490	Lagoa Salgada; Santa Maria	Pantropical
Malvaceae	*Sidastrum paniculatum* (L.) Fryxell	Santos, L.A.S. 1249; Dantas, A. 162	Santo Antônio; Tibau do Sul	Neotropical
Malvaceae	*Waltheria brachypetala* Turcz.	Dantas, A. 298	São Bento do Norte	northeast
Malvaceae	*Wissadula hernandioides* (L’Hér.) Garcke	Santos, L.A.S. 1240	São José do Campestre	American
Melastomataceae	*Clidemia hirta* (L.) D.Don	Moura, E.O. 21	Macaíba	Neotropical
Melastomataceae	*Tibouchina gardneri* (Naudin) Cogn.	Jardim, J.G. 6295	Serra de São Bento	northeast
Myrtaceae	*Eugenia astringens* Cambess.	Dantas, A. 110	Nísia Floresta	Brazilian
Nyctaginaceae	*Guapira campestris* (Netto) Lundell	Roque, A.A. 1103	São José de Mipibu	Brazilian
Nyctaginaceae	Guapira cf. noxia (Netto) Lundell	Silva, R.E. 05*; Gonçalves, F.B. 497*	João Câmara; Porto do Mangue	Brazilian
Phyllanthaceae	*Savia sessiliflora* (Sw.) Willd.	Gonçalves, F.B. 362*	Macaíba	American
Poaceae	*Andropogon fastigiatus* Sw.	Oliveira, R.C. 1743	Canguaretama	Pantropical
Poaceae	*Aristida ekmaniana* Henrard	Flor, R. 268	Ceará-Mirim	Brazilian
Poaceae	*Aristida recurvata* Kunth	Oliveira, A.C.P. sn	Rio do fogo	American
Poaceae	*Cenchrus echinatus* L.	Dantas, A. 189	Baía Formosa	Pantropical
Poaceae	*Chrysopogon zizanioides* (L.) Roberty	Cestaro, L.A. 97-0014	Natal	American
Poaceae	*Digitaria ciliaris* (Retz.) Koeler	Dantas, R.A. 70	Encanto	Pantropical
Poaceae	*Digitaria horizontalis* Willd.	Dantas, A. 190	Baía Formosa	American
Poaceae	*Gymnopogon fastigiatus* Nees	Souza, E.B. 3520; Silva, A.F. 57	Apodi ; Serra Negra do Norte	American
Poaceae	*Hyparrhenia diplandra* (Hack.) Stapf	Jardim, J.G. 5788	Jucurutu	Pantropical
Poaceae	*Lasiacisdivaricata var. austroAmericana* Davidse	Dantas, A. 116	Nísia Floresta	American
Poaceae	*Piresia leptophylla* Soderstr.	Jardim, J.G. 6251	Baía Formosa	American
Poaceae	*Schizachyrium condensatum* (Kunth) Nees	Dantas, A. 231; Flor, R. 266; 267	Ceará-Mirim; Tibau do Sul	American
Poaceae	*Setaria viridis* (L.) P.Beauv.	Santos, L.A.S. 1271	João Câmara	Pantropical
Poaceae	*Sorghumbicolor var. arundinaceum* (Desv.) de Wet & J.R.Harlan ex Davidse	Oliveira, R.C. 2236; 2164	Riacho de Santana; Luís Gomes	Pantropical
Pteridaceae	**Adiantum deflectens Mart.	Moura, E.O. 146	São Francisco do Oeste	Neotropical
Rubiaceae	*Mitracarpus baturitensis* Sucre	Moura, E.O. 138; 212; 213	Carnaubais; Parelhas	Brazilian
Rubiaceae	*Mitracarpus longicalyx* E.B.Souza & M.F.Sales	Silva, A.F. 119A	Rio do fogo	northeast
Sapindaceae	*Allophylus quercifolius* (Mart.) Radlk.	Santos, L.A.S. 1232	São Tomé	northeast
Selaginellaceae	**Selaginella convoluta (Arn.) Spring	Silva, A.F. 68	Cruzeta	Neotropical

**Table 2.** Distribution patterns of species treated here as new botanical records for Rio Grande do Norte state, Brazil.

Distribution pattern	Number of species
Neotropical	21
American	19
Brazilian	14
Pantropical	10
Northeast Brazil	7

### Distribution, phytogeographical domain, and habitats for each new species recorded in RN

AMARANTHACEAE

*Froelichia humboldtiana* (Roem. & Schult.) Seub.

This species occurs in Brazil and Venezuela (Funk et al. 2007, Marchioretto et al. 2005). Inside Brazil it occurs in AL, BA, CE, GO, MG, PB, PE, and PI states in the Caatinga phytogeographical domain (Marchioretto et al. 2005, Marchioretto 2015). In RN, it inhabits anthropic areas with sandy and stony soils.

ANACARDIACEAE

*Spondias purpurea* L.

This species is widely distributed from North and Central America to Brazil, occurring in dry or semi-deciduous forests (Mitchell and Daly 2015). In Brazil, it occurs in AC, AM, BA, and MS states (Mitchell and Daly 2015). In RN, it is recorded from coastal regions with sandy soils.

ASTERACEAE

*Stilpnopappus laiseae* R.Barros & R.Esteves

This species is only known from PI state in Brazil occurring in Caatinga areas (Barros and Esteves 2004) and BA (Loeuille 2015). In RN, it inhabits anthropic areas with shallow grounds or stony soils.

BURSERACEAE

*Protium heptaphyllum* (Aubl.) Marchand

This species has a Neotropical distribution (Pirani 2003). In Brazil, it is widely distributed except in the south region (PR, RS, SC) and in a few states of Northeast (PI, PB, RN). It occurs in the Amazon Rainforest, Caatinga, Central Brazilian Savanna and Atlantic Rainforest (Daly 2015). In RN, it inhabits coastal areas with sandy soils.

CACTACEAE

*Melocactus ernestii* Vaupel

Endemic to Brazil, it is distributed in AL, BA, MG, PB, PE, and SE states and in Caatinga and Atlantic Rainforest (Zappi et al. 2015). In RN, it inhabits rock outcrops.

CELASTRACEAE

*Maytenus acanthophylla* Reissek

This species is endemic to Brazil, where it occurs in BA and MG states where it grows in Caatinga (Lombardi et al. 2016). In RN, it was collected in coastal seasonal forested areas.

CYPERACEAE

*Becquerelia cymosa* Brongn.

This species occurs from Nicaragua and Costa Rica in Central America, Trinidad and Tobago and the Guianas to Brazil in South America (Gómez-Laurito 2003). Previously unknown from DF, GO, MS, PI and RN states where it grows in Amazon Rainforest, Central Brazilian Savanna and Atlantic Rainforest (Alves et al. 2015). In RN, it was collected in seasonal forest in Atlantic Rainforest and Caatinga areas.

*Eleocharis flavescens* (Poir.) Urb.

This species is distributed in the United States, Mexico, Central America, Antilles, and South America (Trevisan and Boldrini 2008). In Brazil, it is widely distributed in the northeast (BA, CE, PB, PE), southeast (MG, RJ, SP) and south (PR, RS, SC) except in AL, SE, and ES states. It occurs in Caatinga and Atlantic Rainforest domains (Alves et al. 2015). In RN, it is found in wetland Atlantic Rainforest areas within restinga.

*Eleocharis maculosa* (Vahl) Roem. & Schult.

This species is widely distributed in the Americas from Central America to South America (Trevisan and Boldrini 2008). In Brazil, it is known for BA, CE, ES, MG, PA, PE, PR, RJ, RR, RS, SC, and SE states. It is widely distributed in all phytogeographical domains (Amazon Rainforest, Caatinga, Central Brazilian Savanna, Atlantic Rainforest, Pampa, Pantanal) (Alves et al. 2015). In RN, it occurs in restinga.

*Eleocharis montana* (Kunth) Roem. & Schult.

This species is distributed from United States to South America including Antilles (Trevisan and Boldrini 2008, Silveira and Longhi-Wagner 2008). It occurs in BA, DF, ES, GO, MG, MS, MT, PE, PR, RS, SC, and SP states, where it grows in Caatinga, Central Brazilian Savanna, Atlantic Rainforest and Pampa (Alves et al. 2015). In RN, it occurs in seasonal wetland areas in Caatinga.

*Rhynchospora caracasana* (Kunth) Boeckeler

This species is distributed in Brazil, Bolivia, Suriname, Guyana and Venezuela (Strong 2006). In Brazil, occurs in BA, CE, DF, MG, and PE states and it is found in Caatinga and Central Brazilian Savanna (Alves et al. 2015). In RN, it was collected in Caatinga areas.

*Rhynchospora gigantea* Link

This species is distributed from Mexico, Central America to Brazil in South America (Guaglianone 2001). In Brazil, it occurs in AL, BA, ES, PB, PE, PR, RJ, RS, SC, SE, and SP states, where it grows in Amazon Rainforest, Caatinga, Central Brazilian Savanna and Atlantic Rainforest (Alves et al. 2015). In RN, it was collected in riparian forest.

*Scleria macrophylla* J.Presl & C.Presl

This species is distributed from Mexico to Brazil including Antilles (Gómez-Laurito 2003). In Brazil, it occurs in BA, DF, GO, MA, MG, MS, MT, PE, PI, RO, and TO states. It is found in Amazon Rainforest, Central Brazilian Savanna and Atlantic Rainforest (Alves et al. 2015). In RN, it was recorded in riparian forest.

EUPHORBIACEAE

*Bernardia sidoides* (Klotzsch) Müll. Arg.

This species is widely distributed from North America to Brazil (Govaerts et al. 2000). In Brazil, it occurs in BA, PE, and MT states, growing in Caatinga and Central Brazilian Savanna (Cordeiro and Secco 2015). In RN, it occurs in shrubby Caatinga with sandy soils and rocky outcrops.

*Croton campestris* A. St.-Hil.

This species occurs in Bolivia and Brazil (Forzza et al. 2010, Jørgensen et al. 2014). It has been previously recorded in AL, BA, CE, DF, ES, GO, MG, MS, PB, PE, PI, PR, RJ, RS, and TO states, where it grows in Amazon Rainforest, Caatinga, Central Brazilian Savanna and Atlantic Rainforest (Cordeiro et al. 2015a). In RN, it occurs in Caatinga and secondary forest with clay soils.

*Ditaxis malpighiacea* (Ule) Pax. & K. Hoffm.

This species is endemic to Brazil and it is only recorded for Al, BA, PI, PE, and PB states, in Caatinga domain (Lucena and Alves 2009, Steinmann 2015). In RN, it was recorded in Caatinga areas.

*Manihot esculenta* Crantz

Native of South America and originated in the Amazon but widely distributed as a cultivated plant (Olsen and Schaal 1999). It has been previously recorded in Brazil for AC, AL, AM, AP, BA, CE, DF, GO, MA, MT, MG, PA, PE, PI, RO, and SP state, in Amazon Rainforest and Caatinga (Cordeiro et al. 2015b). In RN, it was collected in Caatinga and anthropic agricultural areas.

FABACEAE

*Bauhinia dubia* G. Don

Species endemic to Brazil where it is found in AM, CE, MA, PA, PI, and TO states, in Amazon Rainforest and Central Brazilian Savanna (Vaz and Tozzi 2003, Vaz 2015). In this study it was recorded in shrubby Caatinga.

*Calliandra depauperata* Benth.

Endemic to Brazil and previously recorded in BA, CE, PE, and PI states, in Caatinga (Souza 2015). In RN, it occurs in coastal areas with stony soils and shrubby caatinga.

*Calliandra sessilis* Benth.

This species occurs only in Brazil. It has been reviously recorded in BA, CE, MA, MT, MG, PA, PE, and PI states (Souza 2015). It occurs in Amazon Rainforest, Caatinga and Central Brazilian Savanna (Souza 2015). In RN, it was collected in transitional areas between Atlantic Rainforest and Caatinga, and between Atlantic Rainforest and restinga.

*Inga vera* Willd.

*Inga vera* is widely distributed from Mexico to Argentina (Zamora 2010). In Brazil, it is widely distributed so far not recorded only in Al and SE. It occurs in Amazon Rainforest, Central Brazilian Savanna, Atlantic Rainforest and Pantanal phytogeographical domains (Garcia and Fernandes 2015). In RN, it was collected sterile in Caatinga areas.

LECYTHIDACEAE

*Eschweilera ovata* (Cambress.) Mart. ex Miers

This species is known from Brazil in AL, AP, BA, ES, MA, MG, MT, PA, PR, PE, and SE state and Amazon and Atlantic Rainforest domains (Smith et al. 2015). In RN, it was recorded in coastal Atlantic forest areas.

MALVACEAE

*Ayenia erecta* Mart. ex K.Schum.

This species is endemic to Brazil and recorded only in PI state in Caatinga domain (Esteves 2015a). In RN, it was collected in Caatinga with sandy soils and also in anthropic areas.

*Ceiba glaziovii* (Kuntze) K.Schum.

This species is endemic to Brazil (Gibbs and Semir 2003). It is distributed in BA, CE, PB, and PE states in Caatinga, Central Brazilian Savanna and Atlantic Rainforest (Duarte 2015a). In RN, it was recorded in shrubby or forested Caatinga.

*Helicteres guazumifolia* Kunth

*Helicteres guazumifolia* occurs from Mexico to Brazil, in the states of BA, MT, PE, PI, RO, and SE (Cristóbal 2001, Esteves 2015b). In RN, it was collected in transitional areas of Caatinga and Atlantic Rainforest with stony soils.

*Herissantia crispa* (L.) Brizicky

This species is recorded from United State to Argentina (Alves et al. 2011). In Brazil, it occurs in AL, BA, PE, and SE states, in Caatinga and Central Brazilian Savanna (Bovini 2015a). In RN, it was collected in Caatinga with sandy soils.

*Malachra fasciata* Jacq.

This species occurs from Mexico to Bolivia in South America (Fryxell 2007). In Brazil, it has been recorded in BA, MA, MG, PE, and RJ states, in Amazon Rainforest, Central Brazilian Savanna and Atlantic Rainforest (Esteves 2015c). In RN, it was recorded in coastal areas.

*Melochia tomentosa* L.

This species is distributed from United States to Paraguay (Goldberg 1967, Rondón 2007). In Brazil, it occurs in AL, BA, CE, MT, MS, PB, PE, and PI states, in Caatinga, Central Brazilian Savanna and Atlantic Rainforest (Esteves 2015d). In RN, it was collected in shrubby and forested Caatingas.

*Pavonia cancellata* (L.) Cav.

It is distributed from Mexico to Brazil (Esteves and Krapovickas 2009). In Brazil, it occurs in AL, AM, BA, DF, CE, ES, GO, MA, MG, MT, MS, PA, PE, PI, PB, RJ, SE, and SP states, in Amazon Rainforest, Caatinga, Central Brazilian Savanna and Atlantic Rainforest (Esteves 2015e). In RN, it occurs in anthropic areas and stony soils.

*Pseudobombax marginatum* (A.St.-Hill.) A. Robyns

It occurs in South America (Amorim et al. 2009). In Brazil, it has been recorded in BA, CE, DF, ES, GO, MA, MG, MS, MT, PB, PR, RJ, RO, and SP states (Duarte 2015b). During the NFI we recorded it in Caatinga areas.

*Sida acuta* Burm. f

It has a Pantropical distribution (Krapovickas 2007). In Brazil, it occurs in BA, CE, GO, MA, MG, PE, PI, PA, and TO states in Amazon Rainforest, Caatinga, Central Brazilian Savanna and Atlantic Rainforest (Bovini 2015b). In RN, it was recorded in Caatinga areas with shallow and stony soils.

*Sida ciliaris* L.

This species has a Pantropical distribution (Krapovickas 2007). It is a very polymorphic taxon with questionable delimitation (Fryxell 1985, Krapovickas 2007). In Brazil, it occurs in PE state (Amorim et al. 2009). In RN, it was collected in anthropic areas with clay soils and Caatinga with shallow soils.

*Sidastrum paniculatum* (L.) Fryxell

Widely distributed in the Neotropics (Alves et al. 2011), this species occurs in BA, MG, MT, MS, PB, PE, RJ, and SP states, in Amazon Rainforest and Caatinga (Bovini 2015c). In RN, it was collected in shrubby Caatinga and coastal areas.

*Waltheria brachypetala* Turcz.

This species is endemic to Brazil where it occurs in BA, CE, PE, and PI states in Caatinga (Esteves 2015f). In RN, it was recorded in coastal anthropic areas.

*Wissadula hernandioides* (L. Hér.) Garcke

Widely distributed from United States, Mexico, West Indies, Venezuela, Colombia, Bolivia, Paraguay, Argentina, and Brazil (Bovini and Baumgratz 2016). In Brazil, it occurs in BA, MT, MG, PA, PR, RJ, RR, RS, and SP states and in Amazon Rainforest, Central Brazilian Savanna, Atlantic Rainforest and Pantanal (Bovini 2015d). In RN, it was collected in Caatinga areas.

MELASTOMATACEAE

*Clidemia hirta* (L.) D. Don

This species is widely distributed from Mexico to Brazil (Goldenberg et al. 2005). Previously, it was recorded for all Brazilian states except to RN state. It grows in Amazon Rainforest, Caatinga, Cerrado, Atlantic Rainforest (Michelangeli and Reginato 2015). In RN, it grows in Caatinga areas.

*Tibouchina gardneri* (Naudin) Cogn.

This species is endemic to Brazil, where it occurs in CE and PE states and in Caatinga and Central Brazilian Savanna (Guimarães 2015). In RN, it was recorded in decidual seasonal forest.

MYRTACEAE

*Eugenia astringens* Cambess.

This species is endemic to Brazil, it occurs in BA, ES, PR, RJ, SC, and SP states in Atlantic forest (Sobral et al. 2015). In RN, it was recorded in coastal Atlantic forest areas.

NYCTAGINACEAE

*Guapira campestris* (Netto) Lundell

This species is known only from Brazil in BA, DF, GO, MG, and PI states, growing in Central Brazilian Savanna (Sá 2015). In RN, it occurs in semidecidual seasonal forest.

*Guapira noxia* (Netto) Lundell

*Guapira noxia* is endemic to Brazil, where it occurs in DF, GO, MG, MS, MT and SP state in Campo Rupestre and Central Brazilian Savanna (Sá 2015). In RN, it was collected sterile in shrubby Caatinga with stony soils.

PHYLLANTACEAE

*Savia sessiliflora* (Sw.) Willd.

This species occurs from Mexico, Cuba, Puerto Rico, Hispaniola, Caribbean, Venezuela and Brazil (Webster 1998). In Brazil, is recorded only for northeast (BA, CE, PE, SE) and reported only in Caatinga (Secco et al. 2015). In RN, it was collected in ecotonal areas between Atlantic forest and Caatinga.

POACEAE

*Andropogon fastigiatus* Sw.

This species occurs from Mexico, Central America, Antilles to South America and the Old World (Morales 2003). In Brazil, it is recorded in North, Northeast, Central West and Southeast regions in Amazon Rainforest, Caatinga and Central Brazilian Savanna (Zanin 2015a). In RN, it was recorded in anthropic areas with sandy soils.

*Aristida ekmaniana* Henrard

Species endemic to Brazil where it occurs in BA, DF, GO, MG, PR, and SP states in Central Brazilian Savanna (Longhi-Wagner 1990, 2015). In RN, it was recorded in coastal savanna areas.

*Aristida recurvata* Kunth

American species distributed from Belize, Venezuela, Guayanas, Bolivia to Brazil (Morales 2003). In this latter country, it is recorded for BA, DF, GO, MG, MS, MT, PR, RJ, RR, and SP states. It grows in Amazon Rainforest, Central Brazilian Savanna and Atlantic Rainforest (Longhi-Wagner 2015). In RN, it was recorded in Savanna areas the central portion the state.

*Cenchrus echinatus* L.

Pantropical species (Morales 2003). In Brazil, it occurs in BA, CE, DF, GO, MS, MT, PA, PB, PR, RO, RR, and SC state and in Amazon Rainforest, Caatinga, Central Brazilian Savanna, Atlantic Rainforest, Pantanal (Filgueiras 2015a). In RN, it was collected in coastal Atlantic forest areas.

*Chrysopogon zizanioides* (L.) Roberty

This species occurs from United States to Argentina (Filgueiras 2003a). In Brazil, it occurs in BA, RJ, and SP states and in Amazon Rainforest, Central Brazilian Savanna and Atlantic Rainforest (Filgueiras 2015b). In RN, it occurs in white-sand coastal areas.

*Digitaria ciliaris* (Retz.) Koeler

This species occurs from United States to Argentina and in the Old World (Morales 2003). In Brazil, it occurs in AM, BA, DF, ES, GO, MA, MG, MS, MT, PA, PE, PB, PR, RJ, RS, SC, SE, and SP states in Amazon Rainforest, Caatinga, Central Brazilian Savanna, Atlantic Rainforest, Pampa and Pantanal (Canto-Dorow 2015). In RN,it occurs in Caatinga areas.

*Digitaria horizontalis* Willd.

Distributed from United States, Central America to Argentina (Vega and Rúgolo de Agrasar 2003). In Brazil, it occurs in AC, AL, AM, AP, BA, CE, GO, MA, MS, PA, PE, PB, PR, RJ, SC, SP, and TO states in Amazon Rainforest, Caatinga, Central Brazilian Savanna, Atlantic Rainforest and Pantanal (Canto-Dorow 2015). In RN, it was recorded in coastal areas.

*Gymnopogon fastigiatus* Nees

This species occurs from Central America to Bolivia (Morales 2003). In Brazil, it occurs in AM, DF, GO, MG, MT, MS, RO, and SP states in Amazon Rainforest and Central Brazilian Savanna (Valls 2015). In RN, it occurs in anthropic areas with stony soils.

*Hyparrhenia diplandra* (Hack.) Stapf

This species is worldwide distributed. In Brazil, it is only known from PE state Atlantic Rainforest (Filgueiras et al. 2015a). In RN, it was recorded in Caatinga areas.

Lasiacis divaricata var. austroamericana Davidse

This variety occurs in South America from Ecuador to Argentina (Davidse 2003). In Brazil, it was recorded in BA, CE, ES, MA, MG, PR, SP, and SC states in Caatinga, Central Brazilian Savanna and Atlantic Rainforest domains (Filgueiras et al. 2015b). In RN, it was recorded in coastal areas.

*Piresia leptophylla* Soderstr.

This species is distributed from Colombia, Ecuador to Brazil (Judziewicz et al. 2000, Giraldo-Cañas 2011). In Brazil, it occurs in AM, BA, and PE states in the Amazon Rainforest and Atlantic Rainforest domains (Carvalho and Oliveira 2015). In RN, it occurs in white-sand restinga coastal areas.

*Setaria viridis* (L.) P.Beauv.

This species is widely distributed in the new and old world (Pensiero 2003, Morrone et al. 2014). In Brazil, it occurs in AP, DF, GO, MG, RS, and SP states in Amazon Rainforest, Central Brazilian Savanna and Atlantic Rainforest (Shirasuna and Rodrigues 2015). In RN, it was recorded in Caatinga areas.

*Schizachyrium condensatum* (Kunth) Nees

This species occurs from Mexico, Central American, Caribbean to Argentina in South America (Filgueiras 2003b). In Brazil, it was recorded in BA, DF, GO, MG, MS, MT, PR, RS, SC, and SP states in Central Brazilian Savanna, Atlantic Rainforest and Pampa domains (Zanin 2015b). In RN, it was found in coastal areas.

Sorghum bicolor var. arundinaceum (Desv.) de Wet & J.R. Harlan ex Davidse

This is a cultivated and naturalized species originally from Africa that now is worldwide distributed (Longhi-Wagner 2001, Giraldo-Cañas 2011). In Brazil, previously recorded only from Acre state (Filgueiras and Valls 2015). In RN, it was recorded in river banks with clay soil.

RUBIACEAE

*Mitracarpus baturitensis* Sucre

This species is endemic to Brazil (Souza et al. 2010). Recorded in BA, CE, DF, GO, MG, MT, PI, PB, and PE states in Central Brazilian Savanna and Caatinga phytogeographic domains (Souza 2015). In RN, it was collected in shrubby Caatingas with stony soils or secondary forest.

*Mitracarpus longicalyx* E.B.Souza & M.F.Sales

This species is endemic to Brazil, where it occurs in BA, CE, PE, and PI states, restricted to Caatinga domain (Souza et al. 2010, Souza 2015). In RN, it was recorded in anthropic areas with banana plantation.

SAPINDACEAE

*Allophylus quercifolius* (Mart.) Radlk.

This species is restricted to northeast of Brazil. It occurs in AL, BA, CE, PE, and SE states in Caatinga and Central Brazilian Savanna phytogeographical domains (Somner et al. 2015). In RN, it was recorded in Caatinga areas.

## Discussion

Systematic sampling in the entire state of RN during the NFI covered many municipalities that have seen little or no floristic attention in the past. Before the recent surveys, three municipalities – Natal, Mossoró and Serra Negra do Norte – had the highest botanical collecting effort for RN and 129 municipalities had less than 189 records (including 21 without any collection effort whatsoever) (Silva 2015). The low amount of previous effort is reflected in the high number of new occurrences to RN reported in the present study. The municipalities that were previously sampled were mostly concentrated along the Central Potiguar and Agreste Potiguar mesoregions of RN. This study reports new occurrences from the under-sampled Agreste mesoregion (e.g., Pureza), although it also reports new occurrences from some intensively-sampled regions, such as Mossoró, Serra Negra do Norte and Natal. This finding suggests that the botanical sampling in RN remains insufficient even in areas with the highest floristic efforts, such as the capital of the state and the second largest city, which also hosts an herbarium (Mossoró).

Most new occurrences belong to Poaceae, Fabaceae and Malvaceae. Fabaceae and Poaceae are the most species-rich families reported for the Caatinga (BFG 2015) and most new records are from this domain. Although the Atlantic Forest is the most species-rich ecoregion in Brazil (BFG 2015) the NFI conducted more sampling in the Caatinga than in the Atlantic Forest in RN. Sampling effort was allocated in this manner because Caatinga is geographically predominant in this state (Figure 1), while the Atlantic Forest covers only a narrow strip along the east coast, and its area has been severely reduced. Colombo et al. (2016) observed that increased efforts to sample the flora of RN have resulted in improved knowledge of the flora of the Caatinga. Despite our focus on the Caatinga, we found that two of the three municipalities with the highest number of new occurrences, Ceará-Mirim and Canguaretama, were within the Atlantic Forest. This likely reflects the intrinsic diversity of the Atlantic Forest. We believe the Caatinga is the most under-studied area where future sampling efforts should be focused.

Our sampling efforts improved occurrence data of widely-distributed species in Brazil. Some species were originally absent only in RN state, such as *Clidemia hirta* (Melastomataceae), while others were unknown in several other northeastern Brazilian states, such as *Inga vera* (Fabaceae) (also unknown in Alagoas and Sergipe) and *Amburana cearensis* (Fabaceae), which is categorized as endangered (Americas Regional Workshop 1998). *Amburana cearensis* was recently recorded for RN (Amorim et al. 2016) and currently is only absent in Sergipe. We also improved occurrence data of species with restricted distributions, such as *Stilpnopappus laiseae* (Asteraceae) and *Ayenia erecta* (Malvaceae), which were previously reported only from Piauí, and Sorghum bicolor var. arundinaceum (Poaceae), which was previously known only from Acre. We added occurrence data for a few other species already registered for the RN flora but with few known localities. This is the case of the Cactaceae *Tacinga subcylindrica* and *Brasiliopuntia brasiliensis*, previously known from only one collection in the Açu municipality in 2013. New collections from 2015 onwards considerably expanded the documented occurrence of *T. subcylindrica* in RN (to the municipalities of Equador, João Câmara, Areia Branca and Macau). Only one collection of *B. brasiliensis* was reported in 2001 in the municipality of Macaíba, however, new collections were made from 2011 onwards in the municipality of Ceará-Mirim.

Only seven out of 71 newly-occurring species (~10%) have a distribution that is restricted to northeastern Brazil. These low numbers of endemic species are a general pattern for northeast Brazil flora compared to other regions. According to BFG (2015), only 22.7% of 10,661 species of the northeast Brazil are endemic. Also, most of the area in the northeast is Caatinga, which shows a lower percentage of endemic taxa (19.7% of 4,657, BFG 2015), despite being a unique biome in Brazil.

The results of this NFI indicate that the number of species for RN is still underestimated. However, the knowledge of RN’s flora is growing rapidly. New species have recently been discovered (Sobral 2010, Araújo and Alves 2013, Lourenço et al. 2013, Terra-Araújo et al. 2013, Ribeiro et al. 2015b, Souza et al. 2016) and new occurrences for genera or species have been reported for RN (Versieux et al. 2013a, 2013b, São-Matheus et al. 2013, Magalhães et al. 2014, Amorim et al. 2016, Colombo et al. 2016, Gomes-Costa and Alves 2016). In five years, the number of species records for RN increased by 78% (BFG 2015). To continue this trend, we recommend intensive effort focusing on areas that have not been explored by modern taxonomists (Silva 2015), continuous collection across seasons, investment in training of local taxonomists, and improvements to the infrastructure of herbaria.

We emphasize the importance of collaboration among institutions to improve herbarium collections. Most Brazilian herbaria can be considered small, having less than 20,000 records (Vieira 2015). Currently Reflora hosts 54 collections, out of which 40 can be considered small. In our view, small collections are more prone to difficulties due to limited number of staff and funding, lack of visits from specialists (influencing the quality of the data), fewer type specimens, and curators with an overload of tasks (most of them professors). Lack of recognition within institutions, when an herbarium is regarded as belonging to a lab or to a particular professor, leave many collections vulnerable to loss or damage. By facilitating visits by specialists to study specimens, and funding technical fellowships, we improved the quality of our data and incorporated a valuable collection that otherwise would have been abandoned. The impact of visits from specialists on our floristic list is demonstrated by the number of new records reported for Poaceae and Cyperaceae and also by the merging of Museu Câmara Cascudo collection to UFRN herbarium, revealing new occurrences from specimens collected decades ago. The Museu Câmara Cascudo collection for algae is still awaiting examination by specialists, which will likely lead to more new botanical occurrences to RN.

## Conclusion

The partnership between NFI, Reflora, SiBBr and UFRN Herbarium has advanced knowledge of biodiversity by exploring areas with few botanical records and adding new species records for the Flora of Rio Grande do Norte. The geographical distribution of newly-added species is mostly Neotropical (21 spp.), while fewer (seven spp.) are endemic to northeastern Brazil. From these results, we conclude that species that have not been recorded to date may occur in different habitats, and that the entire state requires additional floristic inventories. Furthermore, we revealed areas that were poorly covered by existing botanical collections. We also recorded new species from areas with relatively high previous effort of collection, indicating that the species richness in RN remains underestimated. Even our collections may contain specimens that should be further analyzed and studied by specialists, who rarely have opportunities to visit small herbaria. Future botanical projects should fill these remaining gaps in collections, particularly focusing on seasonality. Finally, the NFI/Reflora/SiBBr projects in RN improved the UFRN herbarium collection by digitizing all specimens and improved the curation of the collection through exchange of material among institutions, increased visibility of our specimens online, and attention from specialists.
